# Effects of protein restriction on insulin-like growth factor (IGF)-1 in men with prostate cancer: results from a randomized clinical trial

**DOI:** 10.1186/s40364-024-00613-w

**Published:** 2024-07-22

**Authors:** Maria L. Cagigas, Giovanni Fiorito, Beatrice Bertozzi, Andrius Masedunskas, Edda Cava, Francesco Spelta, Nicola Veronese, Valeria Tosti, Gayathiri Rajakumar, Tiana Pelaia, Arnold D. Bullock, Robert S. Figenshau, Gerald L. Andriole, Luigi Fontana

**Affiliations:** 1 Faculty of Medicine and Health, Charles Perkins Center, University of Sydney, Camperdown, Australia; 2 Clinical Bioinformatics Unit, IRCCS Istituto Giannina Gaslini, Genoa, Italy; 3 MRC-PHE Centre for Environment and Health, Imperial College London, London, UK; 4 Department of Medicine, Washington University School of Medicine, St. Louis, MO USA; 5 Unit of Dietetic and Clinical Nutrition, Forlanini Hospital, San Camillo, Rome, Italy; 6 Geriatric Unit, Mater Salutis” Hospital, AULSS 9 ScaligeraLegnago, Verona, Italy; 7 Department of Internal Medicine and Geriatrics, Geriatric Unit, University of Palermo, Palermo, Italy; 8 Division of Urology, Washington University School of Medicine in St. Louis, St. Louis, MO 63110 USA; 9 Department of Endocrinology, Royal Prince Alfred Hospital, Sydney, Australia

## Abstract

**Background:**

Insulin-like growth factor (IGF)-1 and its binding proteins are important in cancer growth, especially in prostate cancer. Observational studies suggest that protein restriction can lower IGF-1 levels. However, it is unclear whether an isocaloric protein-restricted diet affects IGF-1 and IGFBPs in men with prostate cancer.

**Methods:**

In this academic, single-center, parallel-group, prospective, randomized, open-label, blinded end-point trial, 38 consenting overweight (BMI 30.5 ± 5.5 kg/m^2^) men with localized prostate cancer, aged 43–72 years, were randomized (1:1) with permuted blocks to 4–6 weeks of customized isocaloric PR diets (0.8 g protein/kg lean body mass) or their usual diet. Biomarkers influencing cancer biology, including serum IGF-1 and its binding proteins were measured longitudinally.

**Results:**

Contrary to our hypothesis, feeding individuals an isocaloric protein-restricted diet did not result in a significant reduction in serum IGF-1. Moreover, there was no observed increase in serum IGFBP-1 or IGFBP-3 concentration.

**Conclusion:**

These findings demonstrate that protein restriction without calorie restriction does not reduce serum IGF-1 concentration or increase IGFBP-1 and IGFBP-3 in men with localized prostate cancer. Further research is needed to identify dietary interventions for safely and effectively reducing IGF-1 in this patient group.

**Supplementary Information:**

The online version contains supplementary material available at 10.1186/s40364-024-00613-w.

To the editor,

The insulin-like growth factor 1 (IGF-1) axis plays a crucial role in the biology of prevalent cancers, including prostate, breast, and colon cancers [1, 2]. In rodents, calorie restriction (CR) lowers plasma IGF-1 concentration by ~ 40%, a reduction that may contribute to its powerful anti-cancer and anti-aging effects [3]. Unlike in rodents, data from observational and randomized trials show that CR with adequate protein intake significantly lowers insulin and increases IGFBP-1, but does not change IGF-1 levels in humans, unless protein intake is also reduced [4, 5]. However, to the best of our knowledge, no randomized feeding trial so far has formally tested the effects of isocaloric protein restriction (PR) on circulating levels of IGF-1 and IGF binding proteins in men with localized prostate cancer who might benefit the most from a sustained reduction in IGF-1 levels.

In this trial, we evaluated the impact of feeding a customized PR diet on serum IGF-1, IGFBP-1 and IGFBP-3 concentrations independent of calorie intake. Otherwise healthy men (aged 43–72 y) diagnosed with localized prostate cancer, scheduled for a radical prostatectomy in four to six weeks, were randomly assigned to a PR diet or their usual ad-libitum Western-like diet. The prescribed protein intake was ~ 0.8 g/kg of lean body mass measured by dual-energy X-ray absorptiometry. PR participants received customized isocaloric diets to maintain body weight.

## A protein restricted diet without calorie restriction does not affect the IGF-1 *axis*

**Fig. 1:**
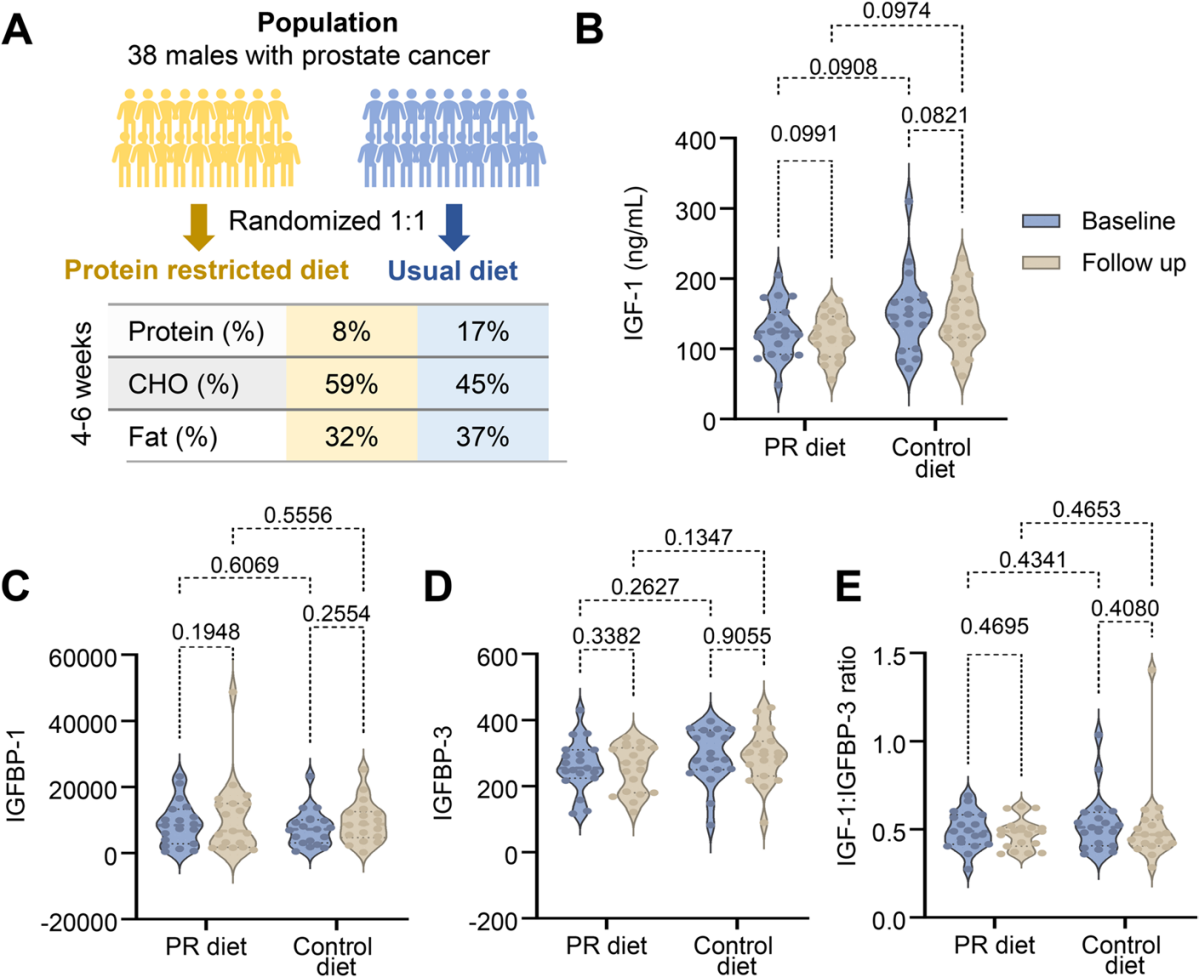
A protein restricted diet without calorie restriction does not affect the IGF-1 axis. **A **Schematic representation of the study. The average macronutrient composition (% of energy) was calculated from provided customized meals (intervention = PR diet) and food diary assessments (control = usual diet). **B**-**E **Fasting blood measurements of IGF-1, IGFBP-1, IGFBP-3 (absolute) and IGF-1:IGFBP-3 ratio (relative) at baseline and at 4–6 weeks follow up. Each dot represents an individual (*n* = 19 per group). Violin plots represent the distribution of the variables. Statistical significance is indicated by p-values calculated using 2-way ANOVA for repeated measurements (baseline and follow-up) and multiple comparisons

**Fig. 2:**
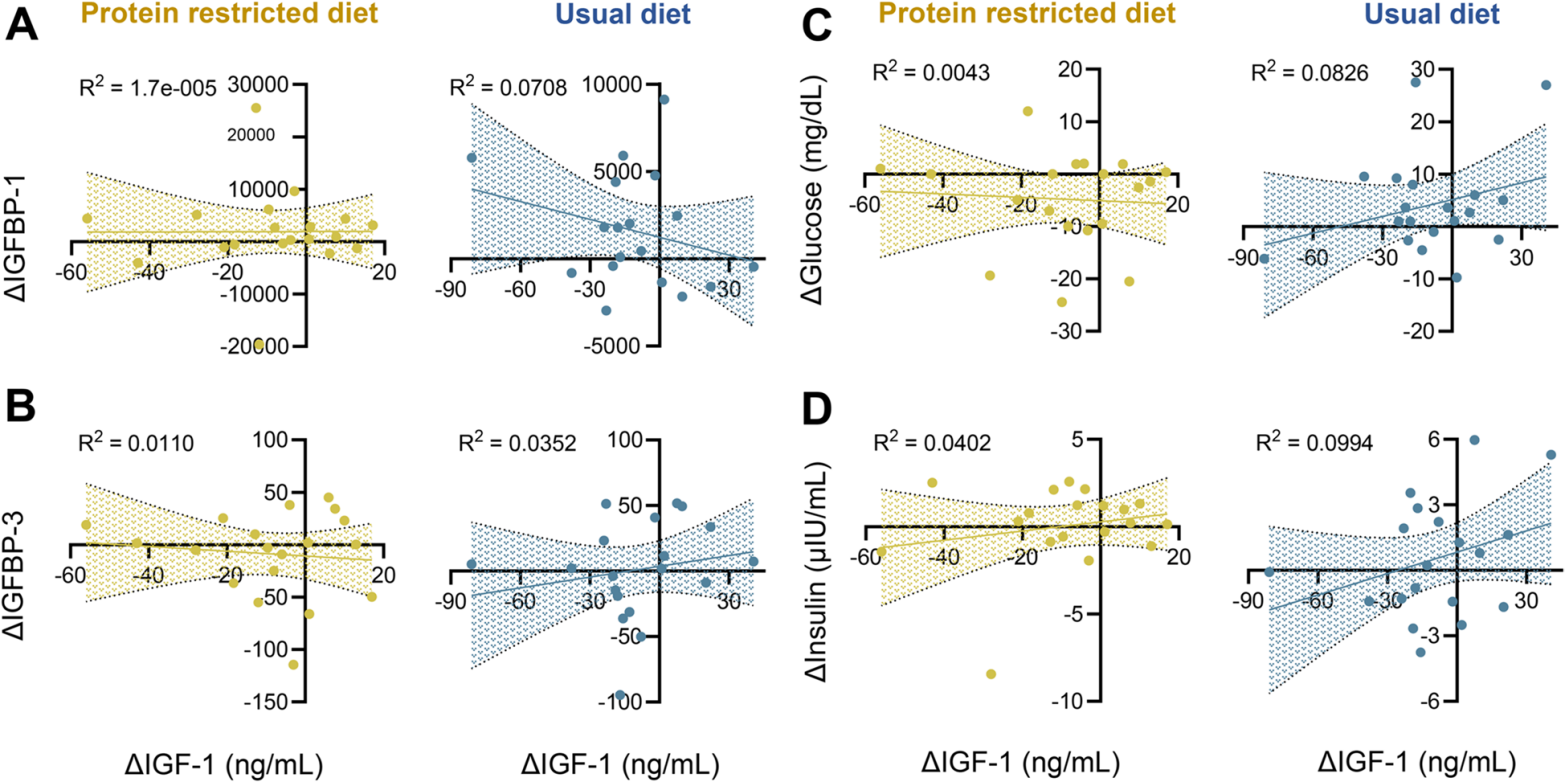
Individual variations in IGF-1 levels are not explained by changes in glucose, insulin, or IGF-binding proteins. **A**-**D** Linear regression analysis between changes in IGF-1 (Δ IGF-1) and Δ IGFBP-1, Δ IGFBP-3, Δ glucose, and Δ insulin for the intervention (yellow) and control (blue) arms. Delta (Δ) represents the value at follow up minus the value at baseline. Each dot represents an individual (*n* = 19 per group). The shaded areas show the 95% confidence bands for the best-fit line. R2 values quantify the strength of the correlation, with R2 < 0.2 suggesting a lack of association

To assess the impact of an isocaloric PR diet on serum IGF-1 and IGFBPs levels, a total of 38 overweight (BMI 30.5 ± 5.5 kg/m^2^) adult males (59 ± 7 years old) adhered to either a calorie-customized 8% protein diet (*n* = 19) or a control diet (*n* = 19) for 43 ± 11 days (Fig. 1a). All meals were provided to maximize compliance. Energy density and macronutrient composition are provided in Supplementary Table 2. Participants in the intervention group consumed 2621 ± 411 kcal/day at baseline and 2856 ± 199 kcal/day during PR (*p* = 0.06), resulting in slight weight loss (101.5 ± 18.8 kg to 98.8 ± 18.6 kg, *p* < 0.0001) despite isocaloric intake, as previously reported [6]. Control participants consumed 2664 ± 611 kcal/day at baseline and 2367 ± 445 kcal/day at follow up (*p* = 0.03), maintaining their body weight (93.2 ± 17.3 kg to 93.0 ± 16.9 kg, *p* = 0.52). At baseline, average serum IGF-1 levels were 126 ± 37 ng/ml in the PR group and 151 ± 55 ng/ml in the control group (between groups p-value = 0.1295). At follow up, IGF-1 levels were 118 ± 30 ng/ml and 141 ± 44 ng/ml, respectively (between group p = 0.9465), showing no evidence of statistical significance and a clinically negligible reduction of 8 ng/ml in the intervention group (Fig. 1b and Supplementary Fig. 1). Additionally, the levels of IGFBP-1, IGBP-3 and IGF-1 to IGFBP-3 ratio did not show evidence of change with isocaloric PR (Fig. 1c-e and Supplementary Fig. 1 and Table 1). We also examined whether fluctuations in glucose and insulin influenced individual IGF-1 outcomes (Fig. 2). PR led to reduced fasting glucose (113 ± 36 to 106 ± 31 mg/dL, *p* = 0.02), unlike the control diet (103 ± 16 to 106 ± 20 mg/dL, *p* = 0.23), whereas insulin remained unchanged in both groups (PR: 7.3 ± 5.3 to 7.4 ± 5.2 µU/mL, *p* = 0.94; control: 6.8 ± 4.2 to 7.3 ± 4.6 µU/mL, *p* = 0.4), as previously reported [6]. None of these variables alone explained the changes in IGF-1 levels in either group (*p* > 0.05 for all; Fig. 2a-d).

## Discussion

This finding challenges the notion that protein intake alone can regulate circulating IGF-1 of IGFBPs levels, as suggested by previous epidemiological studies [7]. Fasting for 3 to 5 days or undergoing severe food restriction (e.g., reducing daily energy intake by over 50%, leading to severe protein restriction) can swiftly and consistently cause a significant decrease in circulating IGF-1 levels in humans [8, 9]. Hence, the absence of an impact on IGF-1 levels from our isocaloric protein restriction cannot be ascribed to a short study duration but rather to the interplay of calorie and protein restriction. Notably, individuals adhering to raw vegetarian diets, maintaining an average daily energy and protein intake of around 1989 kcal and 0.73 g of protein per kg of body weight, respectively, demonstrate lower serum levels of IGF-1 when compared to master athletes of similar BMI [10]. On the other hand, those following high-protein calorie-restricted diets (1772 kcal/day with a protein intake of 1.73 g/kg) did not exhibit lower serum IGF-1 levels, unless there was a concomitant substantial reduction in protein intake [5]. Therefore, a combination of CR and PR is needed to reduce serum IGF-1.

The development of interventions capable of safely and consistently reducing IGF-1 levels, especially in combination with lower insulin, testosterone and inflammation, is of paramount importance, particularly for prostate cancer patients with a high risk of recurrence post-surgery [1, 11]. Approximately 20% to 35% of individuals with prostate cancer who undergo surgery or radiation therapy for localized disease will experience biochemical recurrence [12]. Additional research is required to elucidate what dietary interventions can safely reduce serum IGF-1 levels, particularly in patients with cancers where IGF-1 plays a pivotal role in its development and progression.

### Supplementary Information

Supplementary Material 1.

Supplementary Material 2.

## Data Availability

The data underlying this article are available in the article and in its online supplementary material. Request for additional information can be made to the corresponding author.
