# Supplementary material for: Effects of protein restriction on insulin-like growth factor (IGF)-1 in men with prostate cancer: results from a randomized clinical trial
Source: Biomark Res. 2024 Jul 22;12:68. doi: 10.1186/s40364-024-00613-w (PMC11265162; doi:10.1186/s40364-024-00613-w)
Supplement: Supplementary file 1 — Supplementary Material 1. [file 40364_2024_613_MOESM1_ESM.docx]

**Supplemental data**

**DECLARATIONS**

**Ethics approval and consent to participate.** This study focuses on the secondary outcome (IGF-1 axis) of the trial “Does Protein Restriction Inhibit Prostrate Cancer Growth” (clinicaltrials.gov ID NCT01692587). This was a randomized controlled trial performed at Washington University in St. Louis in the United States, aimed to evaluate the effects of isocaloric PR in otherwise healthy men diagnosed with localized prostate cancer. The study adhered to the Declaration of Helsinki 1975 (1983), and the protocol was approved by the institutional review board of Washington University Medical School, St. Louis, MO, United States (IRB #201011804). Written informed consent was obtained from study volunteers, and oversight was provided by a data and safety monitoring board.

**Availability of data and material.** The data underlying this article are available in the article and in its online supplementary material. Request for additional information can be made to the corresponding author.

**Competing interests.** The authors report no conflicts of interest.

**Acknowledgments and role of funding sources.** This work was supported by grants to L.F. from the Bakewell Foundation, the Longer Life Foundation (an RGA/Washington University Partnership), the National Center for Research Resources (UL1 RR024992), the Australian NHMRC Investigator Grant (APP1177797), and Australian Youth and Health Foundation. G.F. was supported by the Italian Ministry of Health through “Ricerca Corrente'' and “5x1000”. M.L.C. was supported by a Schmidt Science Fellowship. The funding sources were not involved in any form with the findings presented in the study and its submission for publication. The article was not commissioned. No author was precluded access to data.

**Authors' contributions and consent for publication.** Conception and design (L.F.). Trial and sample collection (L.F., B.B., E.C., F.S., N.V., V.T., A.B., R.F., G.A.). Data analysis (G.F., M.C., A.M., V.T.). Interpretation of results and manuscript writing (M.C., L.F.). Review and editing (T.P., G.R.). Funding acquisition (L.F., G.F., M.C). All authors reviewed and approved the final version of the manuscript.

**MATERIALS AND METHODS**

**Study participants.** This study focuses on the secondary outcome (IGF-1 axis) of the trial “Does Protein Restriction Inhibit Prostrate Cancer Growth” (clinicaltrials.gov ID NCT01692587). This was a randomized controlled trial performed at Washington University in St. Louis in the United States, aimed to evaluate the effects of isocaloric PR in otherwise healthy men (aged 43–72 y) diagnosed with localized prostate cancer. Participants were scheduled for radical prostatectomy set no sooner than 6 weeks to ensure a minimum of 4 weeks of intervention. Details of the study design, eligibility criteria, recruitment flowchart, anthropometrics, and participant characteristics were previously reported^22,23^. In brief, after baseline screening, volunteers were randomly assigned in a 1:1 ratio to a control group (n=19) or a PR diet group (n=19) for 4-6 weeks (43±11 days). Men in the control group maintained their usual diet, whereas those in the intervention group consumed an isocaloric PR diet (~0.8 g/kg of LBM) with all meals prepared and provided by the Metabolic Kitchen at the Clinical Translational Research Unit of Washington University. The study adhered to the Declaration of Helsinki 1975 (1983), and the protocol was approved by the institutional review board of Washington University Medical School, St. Louis, MO, United States (IRB #201011804). Written informed consent was obtained from study volunteers, and oversight was provided by a data and safety monitoring board.

**Intervention diet.** Details of the intervention diet, including customization of calorie intake, macronutrient composition, and compliance, were previously reported^22,23^. In brief, to provide participants with isocaloric customized menus, the PR meals were tailored to three calorie levels (2300, 2600 and 2900 calories) to maintain body weight and provided approximately 8% of energy from protein (56-60 grams per day). Compliance was monitored through a daily meal checklist, and energy intakes were estimated using the Harris-Benedict and the Cunningham equations based on individual 4-day food diaries. Men in the control group consumed an ad libitum diet consisting of 45% carbohydrate, 17% protein, and 37% fat (energy %), whereas intervention participants consumed a diet comprising 59% carbohydrate, 8% protein, and 32% fat.

**Biomarkers measurements**. Peripheral blood samples were obtained following an overnight fast at baseline (BL, before any intervention) and at follow-up (FU), after 4-6 weeks of consuming a control or intervention diet). Plasma glucose levels were determined using the glucose oxidase method (YSI Instruments, Fullerton, CA). The measurement of insulin, IGF-1 and IGFBPs was performed in the Core Laboratory for Clinical Studies at the Washington University. The technicians conducting the analyses were blinded to treatment conditions and sample identity. Insulin was measured by commercially available ELISA kits (catalog EZHI-14K, EMD Millipore Corp., St. Charles, MO). IGF-1 and IGFBP-3 were assessed using chemiluminescence with Siemens kits for the Siemens Immulite 1000. IGFBP-1 was performed by ELISA using kits (catalog 11-IGFHU-E01, Alpco, NH, US).

**Statistical analysis.** Data distribution, assessed through the Shapiro-Wilk test for normality and Levene's test for homogeneity, indicated that IGF-1 and IGFBP-3 met normality assumptions, whereas IGFBP-1 and IGF-1:IGFBP-3 ratio did not. All variables fulfilled the homogeneity of variance assumption. Within-group differences between timepoints (BL and FU) for the control and intervention group were calculated using paired 2-tail Student’s t-test and Wilcoxon’s test for parametric and non-parametric data, respectively. Two-way ANOVA assessed the effects of the intervention, timepoint, and their combination. Between-group differences were analysed using independent 2-tail Student’s t-test and Mann Whitney-U test for the difference in means for parametric and non-parametric data, respectively. Significance was set at p < 0.05, with detailed p-values provided in Supplementary Table 1. All values are expressed as means ± SD. To assess associations between delta changes (Δ = FU – BL), simple linear regression between two delta variables (e.g., Δ IGF-1 and Δ glucose) was performed in GraphPad to obtain the best-fit line and 95% CIs. Goodness of fit was represented by R^2^, with R^2^ > 0.2 suggesting an association. Significance was calculated by slope of the best-fit line deviation from zero, with p-values < 0.05 deemed significant.
