# Supplementary material for: Effects of protein restriction on insulin-like growth factor (IGF)-1 in men with prostate cancer: results from a randomized clinical trial
Source: Biomark Res. 2024 Jul 22;12:68. doi: 10.1186/s40364-024-00613-w (PMC11265162; doi:10.1186/s40364-024-00613-w)
Supplement: Supplementary file 2 — Supplementary Material 2. [file 40364_2024_613_MOESM2_ESM.pdf]

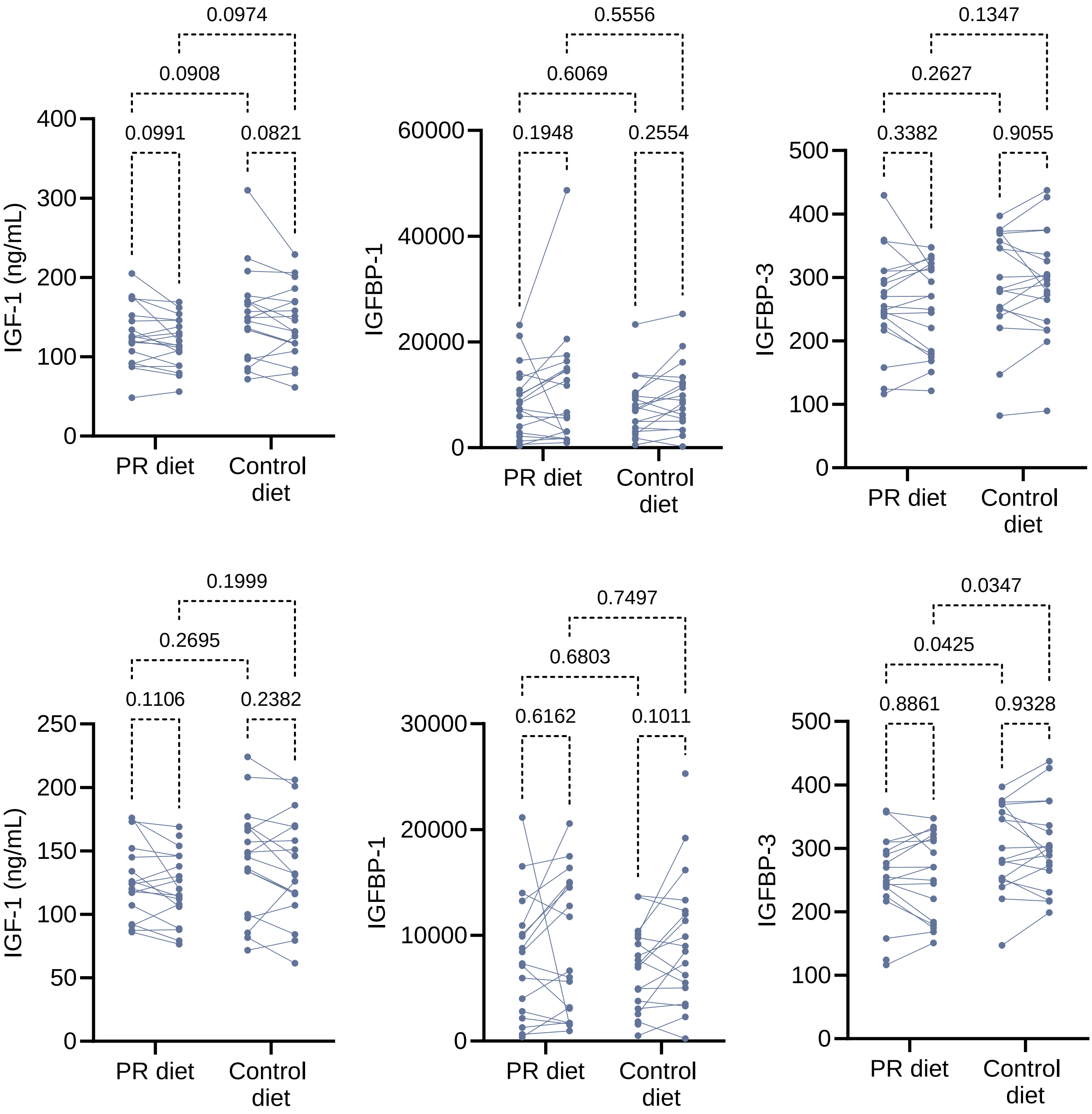

Supplementary Figure 1. Paired fasting blood measurements of IGF-1, IGFBP-1, IGFBP-3 (absolute) at baseline and at 4-6 weeks follow-up. Each dot represents an individual (n=19 per group). Statistical significance is indicated by p-values calculated using 2-way ANOVA for repeated measurements (baseline and follow-up) and multiple comparisons. Results are shown including all data points (top row) and with outliers removed (bottom row). Outliers were defined as those values that deviated further than 2 SDs from the mean.

	Within-group p-value (Baseline vs follow up)		Between-group p-value (mean difference)	ANOVA Intervention effect p-value	ANOVA Timepoint effect p-value	ANOVA Intervention and timepoint combined effect p-value
	Intervention	Control				
**IGF-1**	0.0556	0.1362	0.9465	0.088	**0.019**	0.946
**IGFBP-1**	0.1336	0.1084	0.8819	0.562	0.073	0.971
**IGFBP-3**	0.6226	0.7086	0.4458	0.181	0.551	0.446
**IGF-1:IGFBP-3**	0.3124	0.1232	0.7039	0.422	0.275	0.941

Supplementary Table 1 (P-values associated with Figure 1). Within-group differences between timepoints (BL and FU) for the control and intervention group were calculated using paired 2-tail Student's t-test and Wilcoxon's test for parametric and non-parametric data, respectively. Two-way ANOVA was used to calculate the effect of the intervention, the effect of the timepoint, and the effect of the combination. Between-group differences were calculated using independent 2-tail Student's t-test and Mann Whitney-U test for the difference in means for parametric and non-parametric data, respectively. P-values < 0.05 were considered significant.

	Protein restricted (n=19)	within-group *p*	Control (n=19)	within-group *p*	Among-group *p*
**Energy Intake (kcal/d)**					
Baseline	2621 ± 410.9		2464 ± 611.3		
Follow-up	2856 ± 198.6		2367 ± 444.8 (102.1)		
**Δ Energy Intake**	**235 ± 510.3**	0.06	**-96 ± 347.4**	0.24	**0.03**
**Total Protein (g)**					
Baseline	111.5 ± 16.7		101.0 ± 15.0		
Follow-up	63.8 ± 2.2		95.1 ± 16.2		
**Δ Protein**	**-47.6 ± 16.2**	<0.0001	**-5.9 ± 11.5**	0.04	<0.0001
**Protein (% of energy)**					
Baseline	17.4 ± 2.9		17.1 ± 3.1		
Follow-up	8.3 ± 0.6		16.7 ± 2.5		
**Δ Protein**	**-9.1 ± 3.2**	<0.0001	**-0.5 ± 2.9**	0.49	<0.0001
**CHO (% of energy)**					
Baseline	41.1 ± 7.6		44.3 ± 7.5		
Follow-up	58.9 ± 1.1		44.7 ± 6.5		
**Δ Protein**	**17.8 ± 8.0**	<0.0001	**0.4 ± 7.3**	0.82	<0.0001
**Fat (% of energy)**					
Baseline	38.3 ± 6.8		36.5 ± 7.5		
Follow-up	31.5 ± 1.8		36.6 ± 5.7		
**Δ Fat**	**-6.8 ± 7.3**	0.001	**0.1 ± 6.6**	0.97	0.004

Supplementary Table 2. Dietary parameters for patients on the PR diet. Energy intake and macronutrient composition on Control diet participants, and of all participants at baseline, was assessed from food diaries, while PR diet participants during the trial were provided with customized PR food. Change (Δ) represents the difference between the baseline and follow-up visit. Changes between and within the PR and control groups were tested with analysis of covariance and paired t-tests. Statistical tests were two-tailed, with significance accepted at $p < 0.05$. Errors represent standard deviation. The table was previously published (Fontana et al, Cell Reports, 2016)
